# Pulmonary Consumption

**Published:** 1857-10

**Authors:** 


### *Pulmonary Consumption.*

1. *The influence of Sea Voyages and Warm Climates on the progress of Pulmonary Consumption.* By M. Jules Rochard.—The following conclusions are arrived at on this subject by Mr. Rochard, after a most elaborate investigation of the subject. His paper is published in the "Memoirs of the Academy of Medicine of Paris," for 1856, and is an "ouvrage couronné dans la séance publique du 11 Décembre, 1855."

1. Sea voyages accelerate the progress of pulmonary tuberculization, much more frequently than they retard it.

2. This disease, far from being rare among marines, is, on the contrary, much more common among them than in the land army. It prevails with equal intensity in the hospitals of our ports, in our stations, in our fleets. The "officers de marine," the physicians, the commissaries, all who are afloat, in a word, are subjected to this general law.

3. With rare exceptions, which must be admitted, considering some facts recorded by men of credit, phthisis advances on board ship with more rapidity than ashore.

4. The naval profession should be interdicted, in the most decided manner, to all youths who appeared to be menaced with phthisis.

5. The consumptive can get no advantage from sea voyages, except they be on board under certain special hygienic conditions, and change climate and locality according to the seasons and atmospheric vicissitudes; things which cannot be realized on board of ships with a mission to fulfil. Journeys by land, and prolonged stay in a well-selected country, allow of all the same objects being attained, with much less expense and danger.

6. Warm countries, taken as a whole, exercise an injurious influence on the progress of pulmonary tuberculization, and accelerate its course.

7. Those situated in the torrid zone are especially injurious, and stay there should be formally interdicted to the phthisical. The opinions of the physicians-in-chief of our colonies, and of the English colonies, comparative statistics of colonial and of European regiments in the two sets of countries, the frequency of phthisis in our tropical stations, and in those of England in the same latitudes, and a multitude of special observations, demonstrate this completely, and the examination of each particular locality confirms it.

8. Most hot climates, situated outside of the torrid zone, are equally injurius to the tuberculous. Some points on the confines of this region, and concentrated in a narrow space, are exceptions. This is owing to local conditions. To sojourn in them protects the phthisical from acute affections of the respiratory passages which accelerate the progress of tuberculization, permits a mode of life better adapted to keeping up the general strength, prolongs existence sometimes, and contributes always to a more easy termination of the same.

9. It is in the first stage of phthisis that there is any hope from emigration, and any reason to expect good results from it.

The localities to be recommended to the consumptive M. Rochard divides into four series, according to their respective advantages:

1. Madeira. 2. Hyères, Venice, and Pisa. 3. Rome, Nice, of which the reputation is constantly decreasing. 4. Menton, Villa Franche, Bay of Spezzia, Lake of Como, the Balearic Isles, the Shores of Greece, the North of Egypt, Algeria.—*Abs. Med. Sci., from Edin. Med. Jour.*

2. *Outline of Hygienic Code for the treatment of Consumption.* By Benjamin W. Richardson, M. D., Physician to the Royal Infirmary for Diseases of the Chest. (*The Journal of Public Health, Abst. Med. Sci.*)

In giving the following rules, Dr. Richardson presupposes their general applicability to cases of consumption in all stages of the disease: in the premonitory stage; in the stage where the tubercular disposition is apparent; and in the next stage, when the local mischief is much further advanced. In the last stage even, though hope is lost, many of the rules may still be rigidly followed out with advantage, for by them the course of the disease may be smoothed, and life, perhaps, prolonged.

The Rules are ten in number.

Rule 1.—*A supply of pure air for respiration is the first indication in the treatment of the consumptive patient.*

No cosy room with a temperature of 70°, with every crevice closed, and in an atmosphere in a dead calm and laden with impurities, should be permitted. A temperature from 55° to 65° Fahr. is high enough. The fire, if one be wanted, should be in an open grate, and every arrangement should be made by which the freest possible current of air should be kept circulating through the room. If the patient is cold, he must go near the fire, and, if necessary, poke it, but he must on no account make the room warm by making it close. Dr. Richardson objects very strongly to stoves of all kinds, to heated pipes, and to every other mode of supplying warmth, except an open fire, as by these means the air is made too dry. Among other disadvantages attending the inhalation of too dry air, is hæmoptysis, and a case is mentioned in point. "A gentleman whom I knew, and whose lungs were free from tubercle and other organic disorder, was constantly annoyed and troubled with slight attacks of hacking cough and blood-spitting. He was at a loss to account for the cause. At last he detected that the attacks always commenced when he was at work in his study. With the idea of being very warm and comfortable, and ignorant of the nature of animal heat, he had introduced into a small room a large Burton's stove. To a stranger entering that room when the stove was in action, and the doors and windows snugly closed, the heat and dryness of the atmosphere would have been at once oppressive; but he, a close student, and constantly occupying the room under such conditions, had become accustomed to it as regards external sensation, but caught the mischief effectually in the chest. The cause of the symptoms being explained, the stove was abandoned, and the open fire-grate was again resorted to: the cough and blood-spitting at once disappeared without the administration of any medicine. A few weeks afterwards, thinking that the stove and the cough might only stand in the position of coincidences, our student resumed the use of the stove; and what is more, resumed also, as an effect, the cough and the blood expectoration. This time he became assured that the stove and the cough stood in the relation of cause and effect. The cause was once more removed, and ever since he has remained free of the effect." Free ventilation is especially necessary where gas is used. The bed-room should be large—including, if practicable, not less than 1000 cubic feet of breathing space; and, to prevent any unnecessary contamination of the air, no second person should sleep in the same room, and no light, particularly gas, should be burnt through the night. Two persons in one bed, according to Dr. Richardson, are out of the question under any circumstances. The inclemencies of the weather are not so much to be dreaded as confinement to the house. "I had occasion, some time since," says the author, "repeatedly to remark that if, from a few days' rain, the consumptives under my care were confined to their houses, instead of being able to take the daily out-door breathing always prescribed, the aggravation of the symptoms was always marked and universal. The appetite fell off, the debility became greater, the mind was less bouyant, the local mischief increased." Better go out with respirators and mufflers, under ordinary circumstances, than stay in. A muffler, which a patient may make for a few pence, being as good as any expensive respirator. This is made out of a piece of fine wire gauze, cut oval, so as to cover the mouth and nose, and fixed in the centre of a hankerchief, so that it may be tied on like an ordinary comforter, with the gauze in the centre, for breathing through.

The want of pure air is thought to be an objection to hospitals for consumption. Dr. Richardson admits "that a vast deal of good is, or may be, done at these institutions by the treatment prescribed by the physicians who attend at them, and whose lives are devoted to the study of the disease, there cannot be a doubt. But that it is either physiological, or sound practical treatment, to receive into these buildings consumptive patients, is an assumption I most earnestly dispute. I know the excellent spirit in which institutions of this kind are founded. I am fully aware of the care that is bestowed on the inmates; of the attempts that are made to introduce every hygienic improvement; of the order and cleanliness that prevail; of the kindness of the attendants; of the excellence of the diet-rule; and of the skill of the physicians.

"With all this, it is to me as clear as crystal, that to bring phthisical patients into such institutions is a great charitable mistake. The very care, and waiting-servant attention, that is paid to such of the invalids as are in the first and second stages of the disease, is a cruel kindness. The remedy for them is to encourage and urge them to assist themselves and to exert themselves. Moreover, no kind of hygienic system, carried on in a large building filled with inmates, can make the air of that building in any way equal to the outer air, which it is so necessary that the consumptive person should breathe. Twenty patients, lying in one hospital ward, will throw off per minute into the air of the ward at least three and a half cubic feet of expired and impure gases, rendered in the phthisical the more impure by the pathological condition of the lungs. But the impure air thus exhaled vitiates by its diffusion twenty times its own volume of pure air; so that, in fact, in a ward with twenty patients, there are not less than seventy cubic feet of air spoiled per minute, and rendered unfit for the purposes of life. It may be granted that during the day, when the wards are less full, and many windows are open, and the movements of the inmates are active, the expired air may be fairly disposed of. But take a winter night of twelve hours; consider that in this period of time the twenty patients would, if they exhaled even naturally, vitiate fifty thousand four hundred cubic feet of air, which ought to be removed, and to be replaced by two thousand five hundred and twenty cubic feet of *pure* air for the use of respiration; and then reflect whether it is probable that such a ward can remain during the whole night uncontaminated. For, granting to the twenty patients a breathing-space of twenty-six thousand cubic feet, and even then it would require that the whole of the air in that space should be removed and replaced by fresh air fully twice in the one night. Against this, possibly, the artificial ventilating argumentists will urge that such a feat of ventilation is nothing at all, not worth considering, so easy to be done. M. Grouvelle would probably undertake to effect such interchange eight times in the night, or more; and if he undertook to do it eighty times, and did not succeed in doing it once, it might be difficult to prove the fact against him. But if he would take a strip of paper prepared for ozone, place it in a ward, however artificially ventilated, and place another similar paper in the open air adjoining the ward, it is a mistake if he should not find that there was a striking difference in the process of oxidation in the two localities; and that the great life supporter, oxygen, was in a condition to play a very much more active part in its outdoor than in its indoor work.

"The misfortune of a great hospital, with all its rooms communicating indirectly with each other, that the ventilation is always uncertain. There is, in fact, no properly ventilated space except the great vault of heaven, and no true ventilating power except in the combinations of atmospheric pressure, wind movements, and the force of diffusion.

"If special hospitals for consumptives are to be had, they should be as little colonies, situated far away from the thickly populated abodes of men, and so arranged that each patient should have a distinct dwelling-place for himself. They should be provided with pleasure-grounds of great extent, in which the patients who could walk about should pass every possible hour in the day; and with glass-covered walks overhead, where they could breathe open air, and yet be dry, even if rain were falling. Very expensive such an establishment would be, there is no doubt; but it would, I take it, be infinitely more particularly advantageous to treat ten patients in this manner, than ten tens in a confined brick-and-mortar box, through which of necessity some amount of invisible impurity, some trace of transparent poison-cloud, is constantly floating.

"The strongest argument in favor of consumption hospitals, is, that they receive those members of the community who could not at their own homes afford the same advantages as are supplied to them in the charity. Against this it is to be urged that the patients taken into the consumption hospitals are *not*, in this country at least, in any way to be considered as the representatives of the most needy and destitute sections of the community. These latter go to their last homes in the work-houses, or in their own poverty-stricken dwellings. The classes that fill the hospitals are often many grades above destitution; and are sometimes comparatively wealthy. They have access to a governor, who gives them an admission letter, and they leave their own medical adviser to enter the hospital, not because they cannot find the means to live at home and to be treated at home, but because, catching at every new suggestion offered to them, they set their hearts on getting into the hospital as though it was a certain haven of rescue. In this scramble after admission some of the course succeed; they leave their homes, they enter the hospital, and there the greater proportion of them either die or return back to their friends nearer death than before. A few recover or are relieved; but whether the same result would have occurred, if they had been subjected to the same medical and general treatment out of the hospital—is a question which may be very safely answered in the affirmative."

RULE 2.—*Active exercise is an essential element in the treatment of consumption.*

"Next to a free exposure to air, Dr. Richardson agrees with Drs. Rush, Jackson, and Parrish, in thinking that vigorous exercise is by far the most efficient remedy in the treatment of consumption. "Walking," he says, "is the true natural exercise, and the best, for it brings into movement every part of the body more or less, and, leading to brisker circulation in every part, causes a more active nutrition generally. The extent to which exercise should be carried will vary with the stage of the disease, and temporary accidents may stop it altogether, such, for instance, as an attack of hæmoptysis. But when exercise is advisable, the general rule is to recommend that it be carried out systematically, cautiously, and courageously, and that such exercise should be continued until a gentle feeling of fatigue is felt through the whole muscular system. Violent and unequal exertion of the upper muscles of the body is unadvisable. When restored from the fatigue of one exertion, another should be undertaken, and during the day this cannot be too often repeated. If the day be wet, then the exercise should be effected by walking in a large room, or by engaging in some game, such as skittles, billiards, or tennis.

If, in his waking hours, the consumptive patient can keep himself occupied pretty freely in muscular labor, he secures the best sudorific for his sleeping hours that can possibly be supplied; for as the cause of force is always expended in producing motion or action, so, to use the words of Dr. Metcalf, 'the proximate cause of sleep is an expenditure ef the substance and vital energy of the brain, nerves, and voluntary musclues, beyond what they receive when awake; and the specific office of sleep is the restoration of what has been wasted by exercise.' Cough is very much less frequent in the course of the night in him who has been subjected to exercise in the day; while sleep, when it falls, is more profound, and more refreshing.

"In summer time, when the temperature of the day is high, the morning and the evening time are the best adapted for the periods of outdoor exertion. In the other seasons, mid-day is preferable, as a general rule.

"I have sometimes been asked whether what are called gymnastic exercises are commendable in consumptive cases, and whether swinging is good. My idea on these points is that, in swinging, a person is much more usefully exercised when throwing the swing for his associates' pleasure, than in being swung. There is, in fact, but little faith to be placed in so-called scientific gymnastics. Anything that a man invents to overtop or compete with nature must need be paltry. Brisk natural movement of the limbs is all that the consumptive requires. He need not go out of his way after a sham, in the shape of shampooer; chopping wood is a good gymnastic feat, and playing at skittles is perfect in its way.

"The value of exercise is threefold. First, it checks waste of muscular structures, for muscles left inactive undergo a consumption, without any necessity for lung disorder. Secondly, it diverts the blood from the lungs, causes a more brisk circulation through them, and a more free distribution through the system at large. Thirdly, it induces a more free respiration; more oxygen is taken into the lungs, the body is restored to its vital purposes more surely, and, just in the proportion as this restoration is effected, so is the restoration of disordered function and of disorganized tissue.

"In the performance of muscular exercise let the consumptive never encumber himself, or check the free movements of his body by strappings, loads of clothes, or carrying of weights, and the like. These are but tasks; they lead to unequal exertion in special sets of muscles, and such inequality of expenditure is that which is to be avoided. The treatment of consumption in an hospital is objectionable, again, in regard to exercise. Of what use to the consumptive is an acre or two of airing-ground confined at the back of his hospital? Let him be certain that where the gardener cannot make roses bloom, and peach trees blossom, no doctor can give to the anæmic cheek a permanent color, to a lost function its uses, or to an impoverished body its once healthy power.

"A last consideration on the value of muscular exercise is, that it is eminently useful in keeping the respiratory muscles in a state of active nutrition. For, if to the loss of capaciousness in the lungs to receive air, there is added a daily increasing failure in the muscles by which the acts of inspiration and expiration are carried on, it is clear that a double evil is at work. Now this double evil is most actively presented in consumption. As the respiratory muscles, together with the other muscles, lose their tone, so do the general symptoms of exhaustion increase in severity; sometimes without very marked change in the pathological condition of the lungs. As a sequence, day by day, as the nutrition of these muscles decreases, and as they fail in tonic contractile power, they gain in excitability; so that the irregular spasmodic contractions to which they are subjected in the act of coughing are produced by the merest excitement, and the cough is more frequent as it becomes more feeble.

RULE 3.—*A uniform climate is an important element in the treatment of consumption.*

No particular place is recommended for consumptives, but here is the formula for an hypothetical consumptive Atlantis. "It should be near the sea-coast, and sheltered from northly winds; the soil should be dry; the drinking water pure; the mean temperature about 60°, with a range of not more than ten or fifteen degrees on either side. It is not easy to fix any degree of humidity; but extremes of dryness or of moisture are alike injurious. It is of importance in selecting a locality that the scenery should be enticing, so that the patient may be more encouraged to spend his time out of doors in walking or riding exercise, and a town where the residences are isolated and scattered about, and where drainage and cleanliness are attended to, is much preferable to one where the houses are packed, however small its population may be."

Rule 4.—*The dress of the consumptive patient should be adapted to equalize the temperature of the body.*

Rule 5.—*The hours of rest of the consumptive patient should extend from sunset to sunrise.*

This rule, in Dr. Richardson's opinion, is imperative for many reasons. "First, because in all seasons the actual amount of rest required by the natural man is pointed out with the precision of an astronomical law by the course of the sun. In midwinter men require, for physiological reasons, more sleep than they do at midsummer, and just so much more as is indicated by the difference of night in these two periods. Observe how all animals, left to their own natural instincts, obey this law. Secondly, in our present artificial mode of life, we have to extend the day by the invention of artificial lights. But whenever a man shuts himself up in his closet, and makes a little sun out of his gas-lamp or candle, he is feeding that lamp with part of his own breathing store—the air around him. Worse still, the candle can, no more than the man, live alight without exhaling carbonic acid gas, and thus vitiating the atmosphere. A pound of oil burnt in a lamp produces, in burning, nearly three pounds; and every cubic foot of coal gas, rather more than a cubic foot of carbonic acid. The evil effects of carbonic acid on the lungs have been already described. Thirdly, as an artificial light is, by the mode in which it is produced, of necessity injurious, so, on the contrary, the pure sunlight is of the greatest worth in the acts of vitality. What sunlight does in a physiological way is undetermined; but its general influence has long been known and recognized. Plants banked up from light become blanched, and human beings kept for a long time in dark abodes become the victims of anæmia and scrofula.

"Thus, to fulfil the natural law regulating the times of sleep, to escape from the artificial light, and to obtain the advantage of all the sunlight that can be secured, the consumptive patient should make the sun his fellow workman." But is not this going a little too far? Must the consumptive patient remain eighteen hours in bed in the shortest days of the year, and keep out of bed for the same period in the longest days? and ought the valetudinarian Laplander to remain in bed during his long winter, and to keep up throughout the entire summer? Surely not; and yet, upon this principle, he ought to have no alternative.

Rule 6.—*The occupation of the consumptive patient should be sus-*

*pended if it is indoor or sedentary; but a certain amount of outdoor occupation may be advantageous.*

Among other remarks which naturally arise when speaking upon a rule such as this, is one which deserves considerable attention. It this: "In the case of parents having children of a consumptive tendency, therefore, the greatest care should be taken to obtain for them outdoor employment. But here a serious delusion comes into play. If the child is weakly, the fond parent urges, that it is unfit for hard labor and for outdoor vicissitudes; so it is sent to a tailor or shoemaker, to a clerk's office, a draper's shop, or to some occupation of an indoor character; by this grand, ignorant, and fatal mistake, it is added to the list of the two-thirds who swell the tables of consumption cases."

RULE 7.—*Cleanliness of body is a special point in the treatment of consumption.*

RULE 8.—*Marriage of consumptive females for the sake of arresting the course of the disease by pregnancy is morally wrong, and physically mischievous.*

RULE 9.—*The diet of consumptive patients should be ample, and should contain a larger proportion of the respiratory elements of food than is required in health.*

RULE 10.—*The medicinal treatment of consumption should in the main be of the tonic class.*

There is no doubt, as Dr. Richardson says, that the public expect to be cured by pills and plasters, and not by a series of instructions tending to bring men into obedience with the laws of nature, and therefore much credit is due to him for constructing this decalogue, even though subsequent inquiry may lead him to modify some of its articles.—*N. O. Med. & Surg. Journal.*

